# Building RadiologyNET: an unsupervised approach to annotating a large-scale multimodal medical database

**DOI:** 10.1186/s13040-024-00373-1

**Published:** 2024-07-12

**Authors:** Mateja Napravnik, Franko Hržić, Sebastian Tschauner, Ivan Štajduhar

**Affiliations:** 1 Faculty of Engineering, University of Rijeka, Vukovarska 58, Rijeka, 51000 Croatia; 2 Center for Artificial Intelligence and Cybersecurity, Radmile Matejcic 2, Rijeka, 51000 Croatia; 3 Division of Pediatric Radiology, Department of Radiology, Medical University of Graz, Neue Stiftingtalstraße 6, Graz, 8010 Austria

**Keywords:** Medical data annotation, Data mining, Big data, Feature extraction, Multimodal representation, Unsupervised machine learning

## Abstract

**Background:**

The use of machine learning in medical diagnosis and treatment has grown significantly in recent years with the development of computer-aided diagnosis systems, often based on annotated medical radiology images. However, the lack of large annotated image datasets remains a major obstacle, as the annotation process is time-consuming and costly. This study aims to overcome this challenge by proposing an automated method for annotating a large database of medical radiology images based on their semantic similarity.

**Results:**

An automated, unsupervised approach is used to create a large annotated dataset of medical radiology images originating from the Clinical Hospital Centre Rijeka, Croatia. The pipeline is built by data-mining three different types of medical data: images, DICOM metadata and narrative diagnoses. The optimal feature extractors are then integrated into a multimodal representation, which is then clustered to create an automated pipeline for labelling a precursor dataset of 1,337,926 medical images into 50 clusters of visually similar images. The quality of the clusters is assessed by examining their homogeneity and mutual information, taking into account the anatomical region and modality representation.

**Conclusions:**

The results indicate that fusing the embeddings of all three data sources together provides the best results for the task of unsupervised clustering of large-scale medical data and leads to the most concise clusters. Hence, this work marks the initial step towards building a much larger and more fine-grained annotated dataset of medical radiology images.

## Background

In recent years, research has focused heavily on using machine learning (ML) for medical diagnosis and treatment. Neural networks (NN) and convolutional neural networks (CNN) have shown noticeable results, reducing the effectiveness gap between computer-aided diagnosis (CAD) systems and medical experts [1]. Nevertheless, a major obstacle hindering the development of effective medical models and CAD systems lies in the availability of annotated datasets. Curating and labelling medical datasets requires specialists, making it a time-consuming, expensive, and knowledge-dependent process [2–4]. One viable approach to cope with the data shortage and lack of labelled data is through the Transfer Learning (TL) process  [5, 6].

In the context of NNs, TL means leveraging a model pretrained on one dataset and then fine-tuning it to a specific problem, ultimately requiring fewer data and time compared to NNs with random weight initialisation. One of the most well-known sources of data for constructing pretrained models in the realm of image processing is ImageNet, which comprises hierarchically organised natural images [7]. In a conventional framework, these pretrained models become publicly available once they have been perfected, facilitating other researchers’ attainment of better-performing models within alternative domains in a condensed timeframe. ImageNet pretrained weights of ML model architectures are available for the most popular ML development platforms, such as PyTorch [8], Tensorflow [9], etc.

TL from natural image datasets has become the standard for medical image processing using deep learning [10, 11]. However, when considering medical ML image processing, there are studies suggesting that medical radiology datasets serve as more suitable sources for training pretrained models in comparison to natural image datasets like ImageNet [11–13]. This is understandable because of the semantic disparities in images, caused by a distribution shift between the domains. Additionally, other image properties across different TL domains, such as the number of channels and colour depth, make TL more challenging.

To address this challenge, we explore the possibility of building a large annotated dataset of medical radiology images – RadiologyNET. This dataset aims to serve as a foundational resource for training contemporary model architectures, with the intent of disseminating them publicly through an online platform similarly to ImageNet pretrained weights. The two desired properties of RadiologyNET are: (1) covers major imaging modalities, examination protocols, and anatomical regions, and (2) uses a large number of visually distinct classes (i.e. fine-grained image labelling). To accomplish this, we have gathered a large dataset of medical radiology images (and accompanying textual diagnoses) from the Picture Archiving and Communication System (PACS) at the Clinical Hospital Centre (CHC) Rijeka. Ethical approval for conducting this research was obtained from the competent Ethics Committee.

In accordance with current trends in foundation models training [14], the image annotation is performed using unsupervised learning techniques from available multimodal data sources: images, Digital Imaging and Communications in Medicine (DICOM [15]) tags and narrative diagnoses. This approach aims to generate annotations representing clusters of semantically similar images while segregating pairs of images with disparate semantic content. As a result, the annotations will be assigned to data points with similar properties rather than just similar visual concepts. The described method was inspired by a similar approach proposed in a study by Guo et al. [16]. Their study used unsupervised methods such as autoencoders and k-means to extract relevant features and cluster the data, thus facilitating the labelling process for subsequent use with classification algorithms.

Therefore, the primary contribution of this study is to test, present, and explain an automated, unsupervised approach, to annotating a large medical dataset. In this context, the term “to annotate” refers to the process of attaching a label to a data point (and, consequently, an image) based on its semantic similarity to other data points. During the design phase, special attention was given to ensuring the adaptability and scalability of the approach to facilitate potential future refinements to RadiologyNET. This intention yielded the second contribution of our work, which is a comprehensive description of methods and approaches that should be considered when parsing large medical data repositories. It is also worth noting the potential limitations of our work: (1) RadiologyNET cannot be publicly released due to legal constraints and its substantial size ($\approx 13$ terabytes). (2) The current version of RadiologyNET has a relatively small number of distinct classes compared to the ImageNet database; this issue will be addressed in future dataset versions. (3) Due to hardware and time limitations, experimenting with a bigger number of feature-extracting models was not feasible in the conducted study. We intend to explore more feature-extracting models that may surpass the utilised models in future iterations of RadiologyNET.

This paper is structured as follows. In “Methods” section, we describe the characteristics of the individual data sources of our dataset, the utilised preprocessing and feature extraction methods, and the entire experimental setup. A more detailed description of the data extraction and preprocessing is available in the Appendix. The experiments were conducted on a smaller subset of the RadiologyNET dataset due to the computational constraints of the experimental pipeline. In “Results” section, we first present and compare the evaluation results of different feature extraction and clustering setups on this subset. After applying the best solution to the rest of the data, we describe the annotation characteristics of the current version of the RadiologyNET dataset in “RadiologyNET dataset” section, and its utilisation in future models training in “Clustering quality for future models training” section. Finally, in “Conclusion” section, we conclude the research by presenting the reach and limitations of the annotated dataset and how it will be used in the future to build and share pretrained convolutional neural network models of varying architectures.

## Methods

The layout of this section is as follows. First, the used dataset is described in “Experimentation dataset” section. “Data preprocessing and feature extraction” section details the preprocessing and feature extraction techniques for each of the data sources. Finally, “Experimental setup” section gives an overview of the clustering methods and how the effectiveness of the resulting groups was measured.

### Experimentation dataset

From the original dataset, described in detail in Appendix B, a subset of 135,775 DICOM files and adjoined textual diagnoses was sampled. The subset was balanced with regard to available imaging modalities: Computed Radiography (CR), Computed Tomography (CT), Magnetic Resonance (MR), X-ray Angiography (XA) and Radio Fluoroscopy (RF). Each modality had an equally large representation of 27,155 instances to ensure that our findings equally apply to any of the most commonly occurring imaging modalities. The 135,775 sampled DICOM files were linked to 63,160 different medical examinations. Each exam consisted of a single narrative diagnosis and one or more DICOM files, ranging from 1 to 15 DICOM files per exam. The dataset was randomly split into the train, validation and test subsets: $80\%$ of all exams were used for training, $10\%$ was used for validation, and the remainder for testing. The exact subset sizes are provided in Table 1.

**Table 1:** The sizes of train, test and validation subsets

Subset	Exam (diagnoses) count	DICOM file count
Train	50,528 (80.00%)	108,542 (79.94%)
Validation	6,316 (10.00%)	13,596 (10.01%)
Test	6,316 (10.00%)	13,637 (10.05%)
**Total**	63,160 (100.00%)	135,775 (100.00%)

**Fig. 1:**
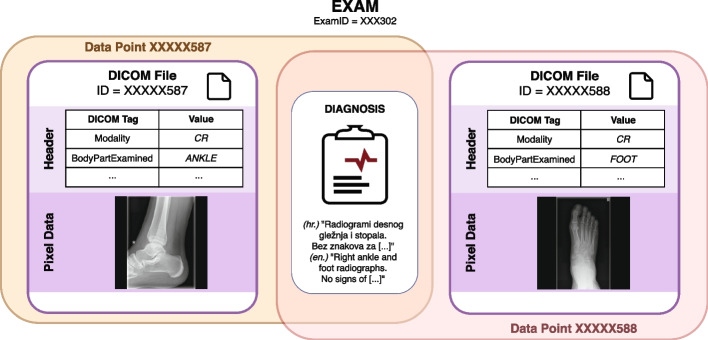
An example of two data points extracted from a single exam, which consisted of two CR images depicting the right ankle and foot. Given that these images were acquired as part of a single examination, they were linked to the same diagnosis, which was written in the Croatian (*hr.*) language. An excerpt from the diagnosis is given in the illustration, along with its English (*en.*) translation

**Fig. 2:**
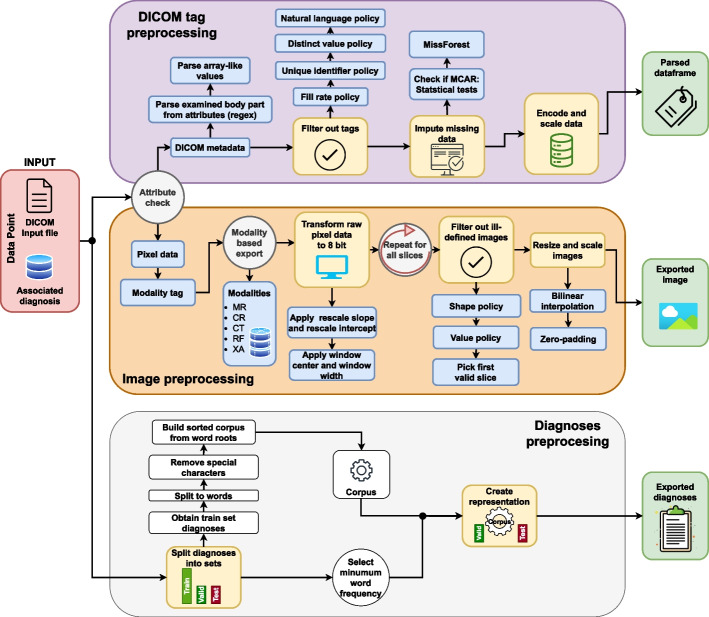
A graphical depiction of the process utilised for exporting images, DICOM tags and narrative diagnoses. Each DICOM file has an associated diagnosis. Each of the three data sources required a distinct preprocessing approach, and each approach had its peculiarities. For example, DICOM tags require additional filtering and the filling in of missing values. On the other hand, images come in different modalities, each requiring a specific approach. Moreover, images are often stored as 12-16 bits arrays, while the monitors and many algorithms support only 8-bit data. Consequently, the images required conversion and additional scaling and resizing. On the other hand, textual diagnoses are written in narrative form. This means that the adverbs, nouns, and verbs had to be stripped to their roots. Moreover, to create a representation of the diagnosis, it was necessary for many methods to build a corpus based on the frequent words in the training set (Table 1). All details concerning data parsing are presented in “Experimentation dataset” section and in the Appendix

As shown in Fig. 1, each DICOM file consists of two main parts: the raw image (pixel data) and the metadata describing the image (DICOM tags located in the file header). Moreover, each DICOM file is accompanied by a narrative diagnosis. All three data sources (i.e. image, tags and diagnosis) were processed independently of each other. The data extraction process is illustrated in Fig. 2. In the remainder of the text, we refer to one recorded instantiation of the three sources’ values as a data point.

In subsequent sections, the preprocessing steps taken for the three data sources to adapt each of them for feature extraction, along with the feature extraction methods, are described. Because of the complexity of these steps, technical details were transferred to the Appendix to make the text easier to follow. Moreover, if the reader is not interested in the technical details concerning data preprocessing altogether, we recommend skipping ahead to “Experimental setup” section. All the choices concerning data preprocessing and feature extraction were determined using training and validation subsets.

### Data preprocessing and feature extraction

The goal of the data preprocessing step for each of the three data sources (tags, images, and diagnoses) was mainly to filter out unuseful data, such as blank DICOM tags, or to discard uninformative data, such as black CT slices.

The goal of feature extraction for each of the three data sources was to identify and extract the most significant and informative patterns from the data. To be precise, the objective was to transform high-dimensional data into low-dimensional embeddings, which could then be fed as input into clustering algorithms. Because there was no reliable ground truth, the methods relied solely on unsupervised feature extraction techniques.

Notably, the best hyperparameter values for all three data sources were chosen based on clustering results obtained on the validation subset. The starting values for each of the hyperparameter spans (where applicable) were selected based on the best practice found in various relevant literature (i.e. papers proposing utilised methods), and are shown in Table 2. The process of preprocessing and feature extraction is depicted in Fig. 2, while the clustering process will be described in “Experimental setup” section. The following subsections discuss each data source’s preprocessing and feature extraction in greater detail.

**Table 2:** Explored hyperparameter value ranges for DICOM tags, image and diagnosis feature extraction. The values were originally taken from related work, and then refined empirically

Data Source	Feature extractor(s)	Hyperparameter	Tested values
DICOM tags	AE	Learning rate	$10^{-2}, 10^{-3}, 10^{-4}, 10^{-5}$
		Bottleneck size (embedding size)	32, 64, 50, 75, 100
		Layer sizes	$1^{st}$: 300, 250, 200, 512, 256
			$2^{nd}$: 200, 150, 125, 100, 256, 128
			$3^{rd}$: 150, 125, 100, 75, 128, 64
	PCA	Solver	LAPACK [17], ARPACK [18], randomised [19]
		Number of components (embedding size)	32, 64, 50, 75, 100
Images	CAE, U-Net, AttU-Net, R2U-Net	Learning rate	$10^{-4}, 10^{-5}, 10^{-6}, 10^{-7}$
		PCA solver	[Not applied], LAPACK, ARPACK, randomised
		PCA number of components (embedding size)	[Not applied], 100, 500, 1000, 10000
Diagnoses	BOW, TF-IDF, PV-DM, PV-DBOW [20]	Minimum word frequency	5, 10, 50, 100, 500, 1000, 2000, 3500, 5000, 10000
	PV-DM, PV-DBOW	Embedding size	10, 25, 50, 100, 250, 500, 1000
		Window size	5, 7, 10
		Number of epochs	25, 50, 100

#### DICOM tags

The in-depth analysis of available DICOM tags identified several problems: finding useful DICOM tags, parsing tags with multiple values, and handling missing data, with the latter being the most prominent issue. Dropping features with missing data can lead to the loss of valuable information, while inadequate handling of missing data can lead to confounded results [21]. The steps in tackling each of these problems are outlined in the next paragraph, while an in-depth description is given in Appendix C. The entire process is also illustrated in Fig. 2.

**Fig. 3:**
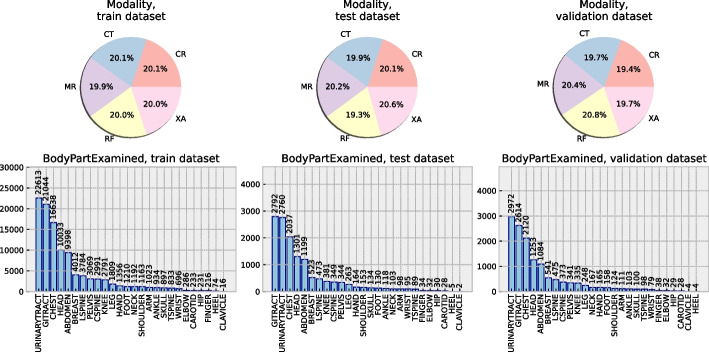
The distribution of image modalities (top) and examined body parts (bottom) in each subset of the used dataset is approximately equal

We intended to use the *BodyPartExamined* (BPE) tag (which describes the anatomical region shown in the image) for evaluation purposes. Therefore, as the first step, (1) it was deemed crucial to reconstruct missing BPE values as accurately as possible. BPE was empty in $59.4\%$ cases, but this was alleviated by analysing other available DICOM tags. Namely, under advice from the radiologist, a set of regular expressions was written based on which BPE values could be inferred from other DICOM tags. The final distribution of examined body parts across all datasets, coupled with the modality distribution, is shown in Fig. 3. Secondly, (2) some DICOM tags contained array-like values. These were parsed so that each array-like tag was split into multiple single-value tags. After this, (3) filtering of DICOM tags was performed to determine which tags could be of use. A fill-rate threshold was imposed on each tag, and each tag which was non-empty in less than $35\%$ cases was excluded from further use. Any tags containing unique identifiers, natural language, or which had less than two distinct values were dropped as well. Furthermore, (4) analysis of data missingness was performed [21, 22] followed by imputation of missing data using MissForest [23–25]. Finally, (5) categorical variables were one-hot encoded and continuous variables were scaled to fit the range [0.00, 1.00].

**The process of DICOM tag feature extraction** was done using principal component analysis (PCA) [26] and autoencoders (AEs) [16, 27]. An extensive grid search of hyperparameters was performed for each approach. Multiple AEs having differing learning rates, architectures and bottleneck layer sizes were trained. All AE encoders consisted of three dense layers of differing sizes, each followed by a rectified linear unit (ReLU) activation function [28]. A bottleneck layer served as a bridge between the encoders and their counterparts (decoders), with the decoders’ architecture being fully symmetrical with respect to the encoder. Across all trained AEs, the maximal number of epochs was 100 with mini-batch size 32, and the chosen loss function was the mean squared error (MSE). If model training showed no loss improvement in 5 consecutive epochs, it was stopped.

Hyperparameter value ranges used in our experiments are shown in Table 2. Initially, when training AEs, all of the learning rates were tested. However, upon a more detailed inspection of the first hundred models, only learning rates $10^{-2}$ and $10^{-3}$ were used due to their superior performance. Moreover, only architectures with gradually decreasing layer sizes were tested.

#### Images

Image preprocessing solves the problem of inconsistent image sizes and pixel depths. Standardising these characteristics is crucial for every ML model to learn effectively, leading to more accurate and consistent results. Several modalities were represented in the provided dataset. Every modality had its own peculiarities that need special addressing when extracting images from the raw pixel data stored in DICOM files. Figure 2 depicts the complete image extraction process.

The raw images found in DICOM files can have a depth between 12 and 16 bits [29]. Before applying feature extractors to images, their pixel depth should be unified, and pixel values normalised. Hence, it is preferable to transform raw images into 8-bit images in a way that causes the least amount of information loss. Namely, the majority of computer displays used for commercial purposes are limited to displaying 8-bit images. Also, most of the ML algorithms are designed to process images where the intensity of pixels falls within the 8-bit spectrum. To achieve this, there are parameters found in DICOM metadata which can be used to appropriately export a DICOM image to an 8-bit image.

The export process requires several parameters stored as DICOM tags. During the data sampling process, we initially verified if all the necessary parameters were available in the DICOM files to determine that the observed DICOM file was eligible for utilisation. The comprehensive procedure for converting raw DICOM pixel data to 8-bit images is outlined in Appendix D. In summary, the image export process consisted of the following: (1) tags *RescaleIntercept* and *RescaleSlope* were read out from DICOM metadata [15] and applied to the raw image; (2) tags *WindowCenter* and *WindowWidth* from DICOM metadata were used to transform the rescaled pixel values into 8-bit data; (3) a *value* policy was implemented to filter out images which were completely single-coloured (e.g. black or white); (4) all images whose shape was erroneous (for example, images stored as 1D vectors) were excluded by applying the *shape* policy; finally, (5) each image was resized to $128\times 128$ pixels using bilinear interpolation, and zero-padding was added where necessary, to preserve aspect ratio. The image size $128 \times 128$ was chosen after careful consideration of the size of the image dataset and limited available processing resources. Additionally, at $128 \times 128$, the level of detail preserved in the images was deemed sufficient to adequately compare their visual similarities. This hypothesis was confirmed by performing a small-scale experiment where the images were resized to $256\times 256$, but the results showed no significant difference.

**To obtain features from images**, multiple neural network architectures commonly used in medical image processing [30] were used. The tested modelling architectures were: convolutional autoencoder (CAE), the original U-Net [31], recurrent residual convolutional neural network based on U-Net (R2U-Net) [32] and U-Net coupled with the attention mechanism (AttU-Net) [33].

The training set was divided into mini-batches of size 32 and, due to a large number of images, validation was performed two times during a single epoch. Adam was used as the optimiser across all models, and the chosen loss function was MSE. All models were allowed to train for 40 epochs but were stopped if validation loss was not reduced in 5 epochs. To see if any further dimensionality reduction could improve clustering results, PCA was applied with an extensive grid search of hyperparameters, as shown in Table 2.

U-Net, AttU-Net and R2U-Net were implemented as described in the original papers [31–33], while the implemented CAE architecture closely follows a similar pattern to the U-Net’s encoder layout. The CAE encoder was comprised of four convolutional layers of $3 \times 3$ kernel size, followed by a ReLU activation function and a $2 \times 2$ max-pooling layer. The layers consisted of 64,128,256 and 512 filters, respectively. The final layer in the encoder was a convolutional layer of 1,024 filters. This was followed by the decoder, which mirrored the encoder’s layout. In all tested models, image features were flattened before performing clustering.

#### Diagnoses

Narrative diagnoses are written in Croatian language and contain information about diseases and patients’ conditions. The Croatian language has several unique aspects concerning word forms, such as seven grammatical cases and different verb suffixes. Two nouns can have the same meaning but are typed differently if they are in different grammatical cases. Hence, the first step was to strip the words to their roots to capture their core meaning better. Namely, we were interested in grouping the diagnoses based on their word meaning similarities, as encoding entire sentences [34] while keeping words intact fell out of the scope of this paper.

Although we are aware of vastly popular generative pretrained transformers (GPT), for this particular problem, we opted for computationally less demanding models. This decision was primarily driven by constraints on method complexity, dataset availability, and computational resources. Given these limitations, experimenting with large neural networks like Bidirectional Encoder Representations from Transformers (BERT) or GPTs [35–37] became unfeasible and was left for future research.

The preprocesing of the textual diagnoses was conducted in several steps. First, (1) all diagnoses from the training subset were split into separate words, with special characters (commas, semicolons, colons...) removed. Then, (2), the words were reduced to their roots using the Croatian stemmer published by Ljubešić et al. [38]. (3) In the training subset, there were a total of 54,790 distinct words from which a word corpus was built. This number encompasses words that appear at least once in the training set’s diagnoses. It was often the case that words present in a small number of instances are actually a result of typographical errors (anomalies) made by physicians who manually write the diagnoses. Hence, in order to enhance the models’ generalisation capabilities, (4) a parameter that regulates the least amount of word occurrences needed for a word to be included in the corpus was introduced. Finally, the pipeline of preprocessing narrative diagnoses is illustrated in Fig. 2.

**To extract feature vectors from narrative diagnoses texts**, the following methods were tested: bag of words (BOW) [20], term frequency-inverse document frequency (TF-IDF) [39], and doc2vec [40]. The utilised methods were selected based on surveys on popular methods for creating embeddings from text (and specifically clinical diagnoses) [41–44].

Hyperparameter value ranges used for processing diagnoses are provided in Table 2. Each method requires a word corpus, i.e. a list of words used to build embeddings by the selected methods. The word corpus was built based on the words present in the training subset. However, for a word to be selected for the corpus, the number of its occurrences had to be greater or equal to the experimentally established threshold *minimum word frequency*.

Each of the selected methods has its advantages and disadvantages; for instance, BOW is the simplest, fastest, and computationally least demanding, but it treats all words equally, no matter how many times they occur in the diagnosis. TF-IDF solves this issue by considering word frequencies but still lacks context representation among the words. Finally, doc2vec methods are based on training a shallow neural network to predict: (1) the following word, using *paragraph vectors: distributed memory* (PV-DM); or, (2) words belonging to the given diagnosis, using *distributed BOW version of paragraph vector* (PV-DBOW). Hence, they can learn the content of a given paragraph by learning the connection between the words present in the paragraph. The input for PV-DM is the paragraph vector (embedding) and context words selected by the window size hyperparameter, while for the PV-DBOW input is only the paragraph vector. The length of learned paragraph vectors (embeddings) is defined by hyperparameter embedding size.

### Experimental setup

Clustering was performed separately on all three sources of data (tags, images, and diagnoses). First, raw data were preprocessed and fed into the described feature extractors, after which the extracted feature embeddings were clustered.

Two clustering algorithms were used: k-means [45] and k-medoids [46]. For k-medoids, two different distance metrics were used: cosine distance and Euclidean distance. Clustering was performed for $\kappa \in \{5, 10, 15, 20, 25, 30, 40, 50, 75, 100, 150\}$ number of clusters. Larger values of the parameter $\kappa$ were experimented with but ultimately omitted from the experimental setup. Namely, during the experiments, several issues, such as a large number of empty clusters, significant overlap between data points from different clusters, or other indications of overfitting, were encountered. It is important to note that clustering algorithms may yield different results based on the initial positions of centroids. To test this potential problem, we conducted 11 independent runs for the best-performing methods, and they showed no statistically significant difference in evaluation metrics.

#### Evaluation metrics

To quantify the quality of the optimal clustering, the main focus was set on cluster homogeneity regarding the imaging modality and the body part examined. Hence, to measure the effectiveness of clustering, homogeneity score (HS) and normalised mutual information (NMI) were calculated for *Modality* and *BodyPartExamined* tags. Both of these metrics have a range [0.00, 1.00], with 0 being the lowest and 1 the highest score. If the imaging modality is denoted as $y_M$, $\hat{y}$ as the predicted cluster label, $I(y_M, \hat{y})$ as mutual information between the two, $H(y_M)$ and $H(\hat{y})$ as their entropy, then NMI regarding modality ($NMI_M$) can be calculated as [47]:

1 $$\begin{aligned} NMI_M = \frac{2 \cdot I(y_M,\hat{y})}{H(y_M) + H(\hat{y})}. \end{aligned}$$

$I(y_M, \hat{y})$ can be calculated as $I(y_M, \hat{y}) = H(y_M) - H(y_M | \hat{y})$, where $H(y_M|\hat{y})$ is the conditional entropy. HS regarding modality ($HS_M$) can be calculated as:

2 $$\begin{aligned} HS_M = 1 - \frac{H(y_M|\hat{y})}{H(y_M)}. \end{aligned}$$

It is important to note that the denominator in the equation for calculating $HS_M$ can never be 0 because the observed subset is not perfectly balanced (the observed “subset is not monotonically pure”). The same process applies when calculating NMI and HS regarding the examined body part ($NMI_B$ and $HS_B$, respectively), with the exception that $y_M$ is replaced by $y_B$, the *BodyPartExamined* tag. The predicted cluster label $\hat{y}$ is always the same.

Finally, overall clustering quality was assessed by calculating the harmonic mean of all four metrics: $HS_B$, $HS_M$, $NMI_B$, and $NMI_M$. This harmonic mean, henceforth referred to as score *S*, provided a comprehensive evaluation of the grouping quality by considering all four metrics simultaneously.

Other than being homogeneous regarding imaging modality and examined body part, optimal clustering results should also exhibit similarities between images and diagnoses. It is expected that all data points within the same cluster will display visible similarities when comparing images and will show that their respective diagnoses carry related information and similar wording. To test this, cosine distances for image and diagnoses embeddings were calculated. The following steps were performed to calculate similarities between images. First, consider there are *k* data points assigned to cluster with index *c*, $0 \le c < \kappa$. For each pair $(i, j), i \ne j$ from cluster *c*, whose images are denoted as $x_I^{(i)}$ and $x_I^{(j)}$ respectively, and their embeddings as $f(x_I^{(i)})$ and $f(x_I^{(j)})$, find the cosine distance as:

3 $$\begin{aligned} d(x_I^{(i)}, x_I^{(j)}) = 1 - \frac{ f(x_I^{(i)})^T \cdot f(x_I^{(j)}) }{ \Vert f(x_I^{(i)}) \Vert \cdot \Vert f(x_I^{(j)}) \Vert }. \end{aligned}$$

The possible number of pairs in cluster *c* is $u^{(c)}=\frac{k}{2}(k-1)$. To find the dissimilarity of images in cluster $D_I^{(c)}$, calculate the mean cosine distance of all pairs from cluster *c*:

4 $$\begin{aligned} D_I^{(c)} = \frac{1}{u^{(c)}} \sum \limits _{i = 0}^{k-1} \sum \limits _{j = i+1}^{k-1} d(x_I^{(i)}, x_I^{(j)}), \end{aligned}$$

and finally, overall image similarities across all clusters were calculated as $D_I = \frac{1}{\kappa } \sum _{c = 0}^{\kappa -1} D_I^{(c)}$.

The same process was applied to get diagnoses similarities $D_{D}$ from diagnoses embeddings. Ideally, $D_{I}$ and $D_{D}$ should be close to 0, or in other words, the distances between embeddings in the same cluster should be as small as possible.

#### Evaluation process

**Fig. 4:**
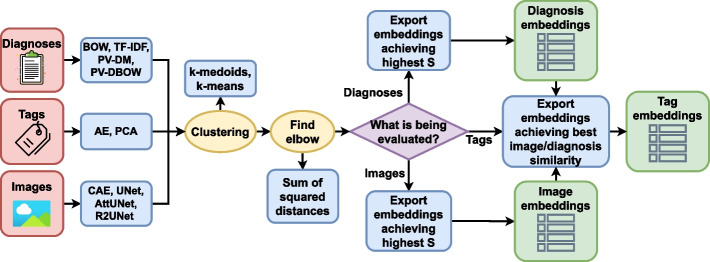
Embedding evaluation pipeline for all three data sources. Diagnoses and images were evaluated by their homogeneity and mutual information of modality and examined body part, which are both tags found in DICOM metadata. On the other hand, DICOM tags were evaluated on the (dis)similarity of diagnoses and images in the obtained groups. Namely, due to the nature of the DICOM standard [15] and the frequent occurrence of modality-specific values, it is more objective to evaluate the DICOM tags based on diagnoses and image embedding (dis)similarity. In short, the images and diagnoses were clustered separately, the best-performing embeddings were chosen and then used to evaluate the performance of DICOM tag clustering. This was done to make a more objective assessment of different data sources’ embeddings

To find the best individual data source embeddings, clustering performance was compared on the validation set across all data sources and all feature extractors. The overall evaluation process is illustrated in Fig. 4. Initially, to find the optimal number of clusters for each of the data sources, elbow method [48] was utilised on the sum of squared distances of data points to their closest cluster centre. The elbow detection is based on the Kneedle algorithm proposed by Satopaa et al. [49]. Having too few clusters could result in a heterogeneous grouping, while having too many clusters might lead to groups that are homogeneous but show evidence of incompleteness [50].

To adequately analyse the clustering results, different sources of data were evaluated using different metrics. The efficiency of DICOM tag clustering was evaluated on image and diagnosis similarities. On the other hand, image and diagnosis clustering was evaluated on how homogeneous the results were regarding the imaging modality and body part examined (based on the metrics $NMI_B$, $NMI_M$, $HS_B$ and $HS_M$).

The best feature extractors for images and textual diagnoses were chosen based on the highest *S* score (found by applying the elbow method). Afterwards, visual similarities $D_{I}$ and textual similarities $D_{D}$ were calculated at the elbow. For this purpose, the best-performing image and diagnosis embeddings from the previous step were used. Finally, to rank the efficiency of DICOM tag feature extraction models, $D_{score}$ was calculated as the harmonic mean of $D_I$ and $D_D$. The primary objective is to create clusters that exhibit the highest degree of data similarity. Therefore, the model obtaining the lowest $D_{score}$ value at the elbow would be selected as the best DICOM tag feature extraction model.

#### Feature fusion

After selecting the best feature extractor for each of the data sources, the resulting embeddings were combined in three ways: direct concatenation, concatenation of cluster-space distances, and concatenation of cluster probability assignments. In each of the approaches, the resulting vector of a single data point *i* was flat, and in the format of $f\left(x^{(i)}\right)=\left[ f\left(x^{(i)}_D\right),f\left(x^{(i)}_T\right),f\left(x^{(i)}_I\right)\right] ^T$, where $f\left(x^{(i)}_D\right)$ is the diagnosis embedding, $f\left(x^{(i)}_T\right)$ is the DICOM tags embedding, and $f\left(x^{(i)}_I\right)$ is the image embedding.

The first approach was to concatenate the raw embeddings from each of the data sources into a single, flat vector. This simple approach was also used in related work, such as [51], where the embeddings were merged from tabular and free-text medical records into a single vector. The second approach was to use cluster-space distances. When clustering a single embedding, distances to each of the cluster centres are computed, and then the point is assigned to the nearest cluster. Embeddings carrying similar information should also have similar distances to each of the cluster centres. Hence, instead of using the extracted embeddings, the computed distances to each cluster centre were used and subsequently concatenated together fusing the data sources. All distances were normalised to fit the range [0.00, 1.00] before concatenation. For ease of reference, this approach will henceforth be referred to as *clusterdists*. The third approach was closely related to the previous one (*clusterdists*), with an additional step of computing the probability assignments for each cluster. The probability of *i*-th data point being assigned to cluster *k* is calculated using the softmax function, assigning higher probabilities to shorter distances:

5 $$\begin{aligned} p_c^{(i)} = \frac{e^{-d_k^{(i)}}}{\sum _{j=1}^{\kappa }e^{-d_j^{(i)}}}, \end{aligned}$$

where $\kappa$ is the number of clusters, and $d_k$ and $d_j$ are distances to *k*-th and *j*-th cluster of the respective source embeddings, respectively. For ease of reference, this approach will henceforth be referred to as *clusterprobs*.

Other methods of feature fusion, such as the approach used by Radford et al. [52], were considered as potential options. However, these approaches were eventually removed from consideration due to constraints posed by hardware limitations and the volume of data involved.

## Results

The results section is structured as follows. First, the results for individual sources are provided in “Optimal embeddings” section. Next, in “Source fusion ablation study” section, the ablation study concerning fusing individual source embeddings is given. The findings concerning the experiments are discussed in “Discussion” section, which also encompasses the description of the final RadiologyNET dataset in “RadiologyNET dataset” section. Lastly, in “Clustering quality for future models training” section, the analysis of possible ways to utilise the clusters for future neural network training is presented.

**Table 3:** Hyperparameter values of best performing feature extraction models (emphasised) for each data source independently and all feature extractors for respective sources covered by our experiments (Table 2)

Data source	Model name	Hyperparameters	Embedding size	Algorithm	Cluster metric	Number of clusters
Images	**CAE**	Learning rate: $10^{-6}$	500	k-means	Euclidean	40
		PCA solver: randomised				
	U-Net	Learning rate: $10^{-6}$	10,000	k-means	Euclidean	30
		PCA solver: randomised				
	AttU-Ne	Learning rate: $10^{-6}$	65,536	k-medoids	cosine	30
		PCA not applied				
	R2U-Net	Learning rate: $10^{-6}$	5,000	k-medoids	cosine	40
		PCA solver: randomised				
Diagnoses	TF-IDF	Minimum word frequency: 1,000	825	k-means	Euclidean	30
	BOW	Minimum word frequency: 10	7,951	k-means	Euclidean	30
	PV-DM	Minimum word frequency: 100	10	k-means	Euclidean	20
		Window size: 5				
		Number of epochs: 50				
	**PV-DBOW**	Minimum word frequency: 50	1,000	k-means	Euclidean	20
		Window size: 7				
		Number of epochs: 50				
DICOM tags	**AE**	$512 \rightarrow 200 \rightarrow 125 \rightarrow 32$	32	k-medoids	cosine	25
		Learning rate: $10^{-2}$				
	PCA	Solver: ARPACK	50	k-medoids	Euclidean	40

**Table 4:** Results for each best performing feature extraction model for images, diagnoses and tag clustering, computed on the validation subset. Best results are emphasised. ± sign delimits the mean from the standard deviation

Data source	Model name	$NMI_B$	$NMI_M$	$HS_B$	$HS_M$	*S*	$D_{D} [\cdot 10^{-2}]$	$D_{I} [\cdot 10^{-2}]$	$D_{score}$
Images	**CAE**	**0.430**	**0.459**	**0.538**	**0.744**	**0.519**	-	-	-
	U-Net	0.401	0.450	0.467	0.673	0.479	-	-	-
	AttU-Net	0.285	0.348	0.328	0.512	0.351	-	-	-
	R2U-Net	0.186	0.206	0.185	0.255	0.205	-	-	-
Diagnoses	TF-IDF	**0.603**	0.493	0.623	0.716	0.598	-	-	-
	BOW	0.529	0.508	0.576	0.788	0.583	-	-	-
	PV-DM	0.544	0.467	0.570	0.689	0.557	-	-	-
	**PV-DBOW**	0.592	**0.569**	**0.634**	**0.77**	**0.633**	-	-	-
DICOM tags	**AE**	-	-	-	-	-	**3.553 ± 2.11**	**3.051 ± 1.01**	**3.283**
	PCA	-	-	-	-	-	3.578 ± 2.019	3.248 ± 1.171	3.405

### Optimal embeddings

As was described in “Data preprocessing and feature extraction” section, ten different models in total were trained for feature extraction: four models for textual diagnoses (TF-IDF, BOW, PV-DM, PV-DBOW), another four models for images (CAE, U-Net, AttU-Net, R2U-Net), and two models for DICOM tags (AE, PCA). Embeddings obtained by each of these extractors were tested on all hyperparameter values and across all clustering setups. In Table 3, we present the best hyperparameter values for each of the four tested models types, while in Table 4, we provide the results for the selected best-performing models.

**Fig. 5:**
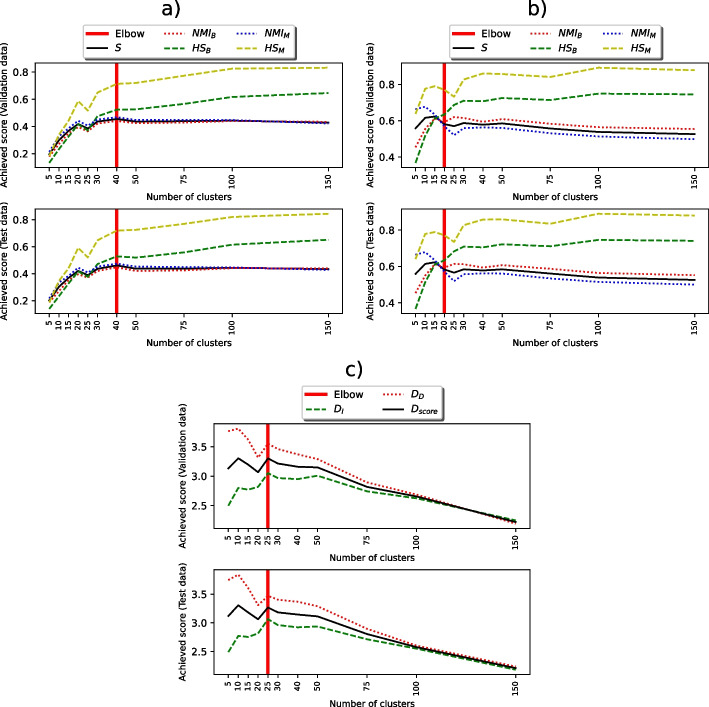
Diagrams showing individual evaluation metrics values on validation (top) and test (bottom) subsets, when clustering optimal image embeddings (CAE, subfigure **a**)), optimal diagnoses embeddings (PV-DBOW, subfigure **b**)) and DICOM tag embeddings (AE, subfigure **c**)). For all the data sources and extractors, the performance is almost identical on both subsets

Results show that CAE is the best-performing model among the image extractors. In terms of modality and examined body part homogeneity, CAE outperformed U-Net, AttU-Net, and R2U-Net. It obtained the highest $HS_M$, $HS_B$, $NMI_M$, and $NMI_B$ on the validation subset, and thus the highest *S* score.

Regarding extractors for narrative diagnoses, PV-DBOW attained the highest $HS_M$, $HS_B$ and $NMI_M$ scores. While its $NMI_B$ was second only to TF-IDF, the overall *S* score shows that PV-DBOW outperformed the other models.

To calculate image and diagnoses distances (and the corresponding $D_{score}$) for DICOM tag evaluation, the best-performing feature extractors from images and diagnoses were used, which were CAE and PV-DBOW, respectively. As it can be seen in Table 4, the best image and diagnosis similarity on the validation subset was achieved using AE.

A more detailed performance of best-performing models’ clustering results is shown in Fig. 5, where Fig. 5a shows CAE performance as the highest scoring image feature extractor, Fig. 5b shows the same for diagnoses (PV-DBOW) and Fig. 5c for DICOM tags (AE). From the shown metrics, it is evident that the models perform nearly the same on the validation and test sets, showcasing that the models did not overfit.

### Source fusion ablation study

Based on the obtained results, the chosen models for feature fusion were: AE for DICOM tags, CAE for images and PV-DBOW for diagnoses. The next goal of our study was to extensively investigate the relation between clustering results and embedding sources included in clustering. An analysis of hyperparameters was performed on the validation set, based on which the best hyperparameter values were chosen and are shown in Table 5, while their respective performance on the validation set can be seen in Table 6. The best hyperparameters were chosen primarily based on their performance regarding the metric *S*, while the metric $D_{score}$ was taken into consideration where the metric *S* was deemed insufficient to adequately distinguish between the best results.

**Table 5:** Hyperparameter values of each best performing model obtained by fusing individual source embeddings. Model performance is shown in Table 6). In this table,*AE* is used to describe DICOM tag embeddings, *CAE* as image embeddings as *PV-DBOW* as diagnosis embeddings

Model name	Combine Method	Embedding size	Algorithm	Clustering metric	Num clusters
[AE]-[CAE]	clusterdists	65	k-means	Euclidean	30
	clusterprobs	65	k-medoids	cosine	30
	embeddings	532	k-means	Euclidean	75
[PV-DBOW]-[AE]	clusterdists	45	k-means	Euclidean	30
	clusterprobs	45	k-medoids	Euclidean	30
	embeddings	1032	k-means	Euclidean	40
[PV-DBOW]-[CAE]	clusterdists	60	k-medoids	cosine	30
	clusterprobs	60	k-medoids	Euclidean	40
	embeddings	1500	k-means	Euclidean	40
[PV-DBOW]-[AE]-[CAE]	clusterdists	85	k-medoids	Euclidean	40
	clusterprobs	85	k-medoids	cosine	40
	embeddings	1532	k-means	Euclidean	50

**Table 6:** Results for each best-performing model for all combinations of data sources, computed on the validation subset. Best results are emphasised for each specific metric utilised. ± sign delimits the mean from the standard deviation. In this table, *AE* is used to describe DICOM tag embeddings, *CAE* as image embeddings as *PV-DBOW* as diagnosis embeddings

Model name	Combine Method	$NMI_B$	$NMI_M$	$HS_B$	$HS_M$	*S*	$D_{D} [\cdot 10^{-2}]$	$D_{I} [\cdot 10^{-2}]$	$D_{score}$
[AE]-[CAE]	clusterdists	0.486	0.652	0.574	0.99	0.631	3.599 ± 1.976	2.663 ± 1.129	3.061
	clusterprobs	0.503	0.68	0.578	0.999	0.647	3.564 ± 2.061	2.759 ± 1.101	3.11
	embeddings	0.462	0.534	0.613	0.928	0.593	3.232 ± 1.787	2.268 ± 0.976	2.666
[PV-DBOW]-[AE]	clusterdists	0.46	0.675	0.532	0.999	0.612	**2.894 ± 2.356**	2.717 ± 1.039	2.802
	clusterprobs	0.516	**0.697**	0.581	**1.0**	0.656	3.385 ± 2.205	2.802 ± 1.045	3.066
	embeddings	0.609	0.563	0.712	0.845	**0.666**	3.228 ± 1.891	2.179 ± 0.863	2.602
[PV-DBOW]-[CAE]	clusterdists	0.373	0.429	0.44	0.651	0.453	2.927 ± 2.038	2.457 ± 0.965	2.671
	clusterprobs	0.425	0.453	0.529	0.732	0.512	3.269 ± 1.878	2.272 ± 1.031	2.681
	embeddings	**0.611**	0.548	**0.713**	0.819	0.657	3.234 ± 1.957	2.163 ± 0.879	2.592
**[PV-DBOW]-[AE]-[CAE]**	clusterdists	0.472	0.616	0.583	0.986	0.618	3.362 ± 2.088	2.587 ± 1.104	2.924
	clusterprobs	0.497	0.639	0.604	**1.0**	0.642	3.483 ± 1.996	2.779 ± 1.134	3.091
	**embeddings**	0.587	0.57	0.707	0.885	**0.666**	2.982 ± 1.688	**2.012 ± 0.783**	**2.403**

DICOM tags and images ([AE]-[CAE]): When observing results given in Table 6 and those in Table 4, it becomes apparent that grouping DICOM tags with images leads to an improvement in the $D_{score}$ compared to using AE alone. Moreover, all three combine methods (*embeddings*, *clusterdists* and *clusterprobs*) yield a higher *S* score than using images alone, exhibiting better modality and examined body part homogeneity.

**Fig. 6:**
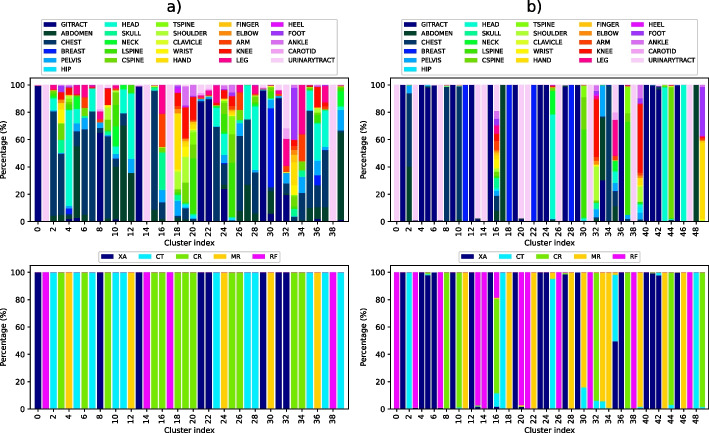
Grouping quality regarding modality and examined body part, when grouping by [PV-DBOW]-[AE]-[CAE] using *clusterprobs* (subfigure **a**)) and *embeddings* (subfigure **b**)) combine methods. In these plots, each bar represents the mixture ratio within a specific cluster. In subfigures **a**) and **b**), the first image (top) shows how homogeneous the clusters are when observing the body part (i.e. how mixed the clusters are with regard to anatomical region), while the second one (bottom) shows the different modalities in each cluster (i.e. how mixed the clusters are with regard to imaging modality)

Diagnoses and DICOM tags ([PV-DBOW]-[AE]): Following a similar pattern to [AE]-[CAE], combining DICOM tags with diagnoses leads to an improvement in the $D_{score}$ compared to using DICOM tags alone. When applying the *embeddings* combination method, the *S* score is higher than the one obtained using just diagnosis (and is the highest obtained overall on the validation subset), with visible improvement, particularly in $HS_B$ and $HS_M$. Moreover, when observing the *clusterdists* and *clusterprobs* methods, the combined approach exhibits a notable increase in modality homogeneity compared to just using diagnoses embeddings. However, there is a trade-off in terms of examined body part homogeneity, as $NMI_B$ and $HS_B$ are lower in the *clusterdists* and *clusterprobs* approaches than the $HS_B$ and $NMI_B$ obtained by diagnoses alone.

Diagnoses and images ([PV-DBOW]-[CAE]): Combining images with diagnoses, particularly using the *embeddings* method, results in the best grouping by examined body part; in this manner, the overall highest $NMI_B$ and $HS_B$ are achieved. The *S* score obtained through this combination is better than the *S* score achieved by using images and diagnoses independently.

Diagnoses, DICOM tags and images ([PV-DBOW]-[AE]-[CAE]): Lastly, when all three data sources (images, DICOM tags, diagnoses) are combined using *embeddings* method, the best overall *S* score is achieved. This score is equal to the *S* score obtained when using [PV-DBOW]-[AE] (diagnoses and DICOM tags) using the *embeddings* approach; however there is a clear difference in $D_{score}$ between them. On the other hand, when all three data sources are combined using the *clusterprobs* method, a perfect score for $HS_M$ is obtained. Figure 6a and b show how different quality of groupings are obtained through these two different combine methods (*clusterprobs* versus *embeddings*).

Finally, the performance of all individual data sources, as well as all feature combinations and combination methods on the test subset is given in Table 7.

**Table 7:** Clustering results on the test subset, when using the best performing models from all data sources and all three feature fusion approaches. ± sign delimits the mean from the standard deviation

Model name	Combine Method	$NMI_B$	$NMI_M$	$HS_B$	$HS_M$	*S*	$D_{D} [\cdot 10^{-2}]$	$D_{I} [\cdot 10^{-2}]$	$D_{score}$
AE (DICOM Tags)	-	-	-	-	-	-	3.471 ± 2.059	3.065 ± 1.107	3.255
CAE (Images)	-	0.447	0.474	0.528	0.719	0.524	-	-	-
PV-DBOW (Diagnoses)	-	0.594	0.571	0.634	0.771	0.634	-	-	-
[AE]-[CAE]	clusterdists	0.494	0.652	0.584	0.992	0.637	3.592 ± 1.941	2.654 ± 1.146	3.053
	clusterprobs	0.507	0.678	0.584	**1.0**	0.65	3.546 ± 2.026	2.769 ± 1.109	3.109
	embeddings	0.465	0.539	0.615	0.933	0.597	3.22 ± 1.711	2.256 ± 0.983	2.653
[PV-DBOW]-[AE]	clusterdists	0.462	0.671	0.536	0.999	0.614	**2.906 ± 2.358**	2.697 ± 1.058	2.797
	clusterprobs	0.523	**0.69**	0.593	**1.0**	0.662	3.364 ± 2.172	2.801 ± 1.09	3.057
	embeddings	0.605	0.561	0.711	0.847	0.664	3.172 ± 1.815	2.179 ± 0.921	2.584
[PV-DBOW]-[CAE]	clusterdists	0.368	0.424	0.434	0.642	0.447	2.913 ± 1.991	2.442 ± 0.967	2.657
	clusterprobs	0.427	0.455	0.532	0.736	0.514	3.279 ± 1.85	2.269 ± 1.039	2.682
	embeddings	**0.611**	0.546	**0.712**	0.816	0.656	3.208 ± 1.92	2.182 ± 0.93	2.598
**[PV-DBOW]-[AE]-[CAE]**	clusterdists	0.478	0.617	0.591	0.989	0.623	3.354 ± 2.096	2.571 ± 1.1	2.911
	clusterprobs	0.499	0.637	0.606	**1.0**	0.643	3.47 ± 1.94	2.787 ± 1.173	3.091
	**embeddings**	0.587	0.571	0.705	0.885	**0.666**	3.024 ± 1.591	**2.011 ± 0.828**	**2.416**

## Discussion

One of the main challenges encountered in presented research on how to approach data annotation involved identifying proper distinct classes, or annotation ontology. First, we explored the utility of applying LOINC/RSCNA Radiology Playbook [53] for guiding the annotation process. After in-depth inspection of the data, it was noticed that the current clinical practice at CHC Rijeka differs significantly from what is proposed in the standard. Similar attempts were made to conjure our own alternative annotation ontology. However, this idea was also abandoned because of other challenges that were difficult to solve, such as region overlapping. As a result, the idea of structuring the annotation process was abandoned, leaving it completely to the unsupervised process.

As can be seen in Table 7, of all the three data sources, image clustering (CAE) exhibited the worst performance regarding modality and examined body part homogeneity. Upon visual inspection of the obtained clusters, it was noticed that although images can be visually similar, they often showcase different body parts captured by the same modality or vice versa; they show the same body part but are captured in different modalities. Different windowing parameters can greatly influence the image as well, an example of which can be seen in cluster 2 shown in Fig. 8. Although all images show a part of the torso, there are significant differences in pixel intensity, which could lead to confounded grouping and a lower $NMI_B$ score. This suggests that images alone do not provide sufficient information for semantically good grouping. However, the results shown in Table 7 indicate that integrating image data into the grouping process reduces the $D_{score}$ and improves the visual quality of the clusters. Thus, although images may not individually possess enough information for optimal semantic grouping, their inclusion contributes to enhanced cluster representation.

Diagnoses (PV-DBOW) showed excellent results regarding the quality of anatomical region grouping. This could be explained by the fact that diagnoses’ wording has a significant focus on the anatomical region being examined by describing illnesses or injuries affecting a specific body part. When used in conjunction with other data sources – as shown in Table 7 – it is evident that the quality of grouping by examined body part is significantly improved. Therefore, it can be inferred that the integration of diagnoses contributes to better anatomical region grouping.

Wherever DICOM tags (AE) were used, there was a noticeable improvement with regard to modality homogeneity (Table 7). When coupled with DICOM tags, images achieved better $NMI_M$ and $HS_M$ results than they did independently. This is visible with diagnoses as well, where $HS_M$ increases when they are joined with DICOM tags. It is evident that DICOM tags contribute to better modality homogeneity, which can be explained by analysing the DICOM standard [15] and observing that DICOM tags often have modality-specific values.

Three different feature fusion approaches were tested, all three showing satisfactory performance. Two alternative methods of feature fusion, namely *clusterprobs* and *clusterdists*, were introduced and tested. To the best of our knowledge, no similar feature fusion techniques have been used before. When compared to the *embeddings* approach, *clusterdists* and *clusterprobs* favoured high modality homogeneity. In particular, three different models shown in Table 7 ([PV-DBOW]-[AE]-[CAE] *clusterprobs*, [AE]-[CAE] *clusterprobs* and [PV-DBOW]-[AE] *clusterprobs*) achieved perfect scores for $HS_M$. Nonetheless, the *embeddings* approach consistently outperformed the others in terms of anatomical region homogeneity. This is especially visible in Fig. 6a and b, where the former shows how it prioritises modality homogeneity, while the latter exhibits superior grouping in terms of the anatomical region.

As is visible in Table 7, some models achieved similar *S* scores. However, between these models there is a clear $D_{score}$ difference, with lower $D_{score}$ meaning that clusters are more visually homogeneous and contain more similar diagnoses, which is favourable. Thus, the approach utilising all three data sources combined using the *embeddings* method can be considered the most efficient for achieving the best grouping results in terms of modality, examined body part, and image/diagnosis similarity.

### RadiologyNET dataset

**Fig. 7:**
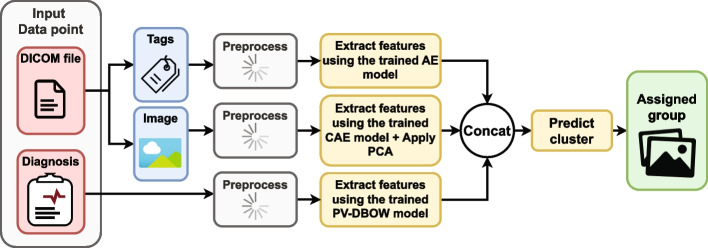
The fully unsupervised labelling algorithm. From an example data point, each data source was processed independently and then fused together to form a single embedding. Afterwards, this embedding was used to assign this data point to a group

**Fig. 8:**
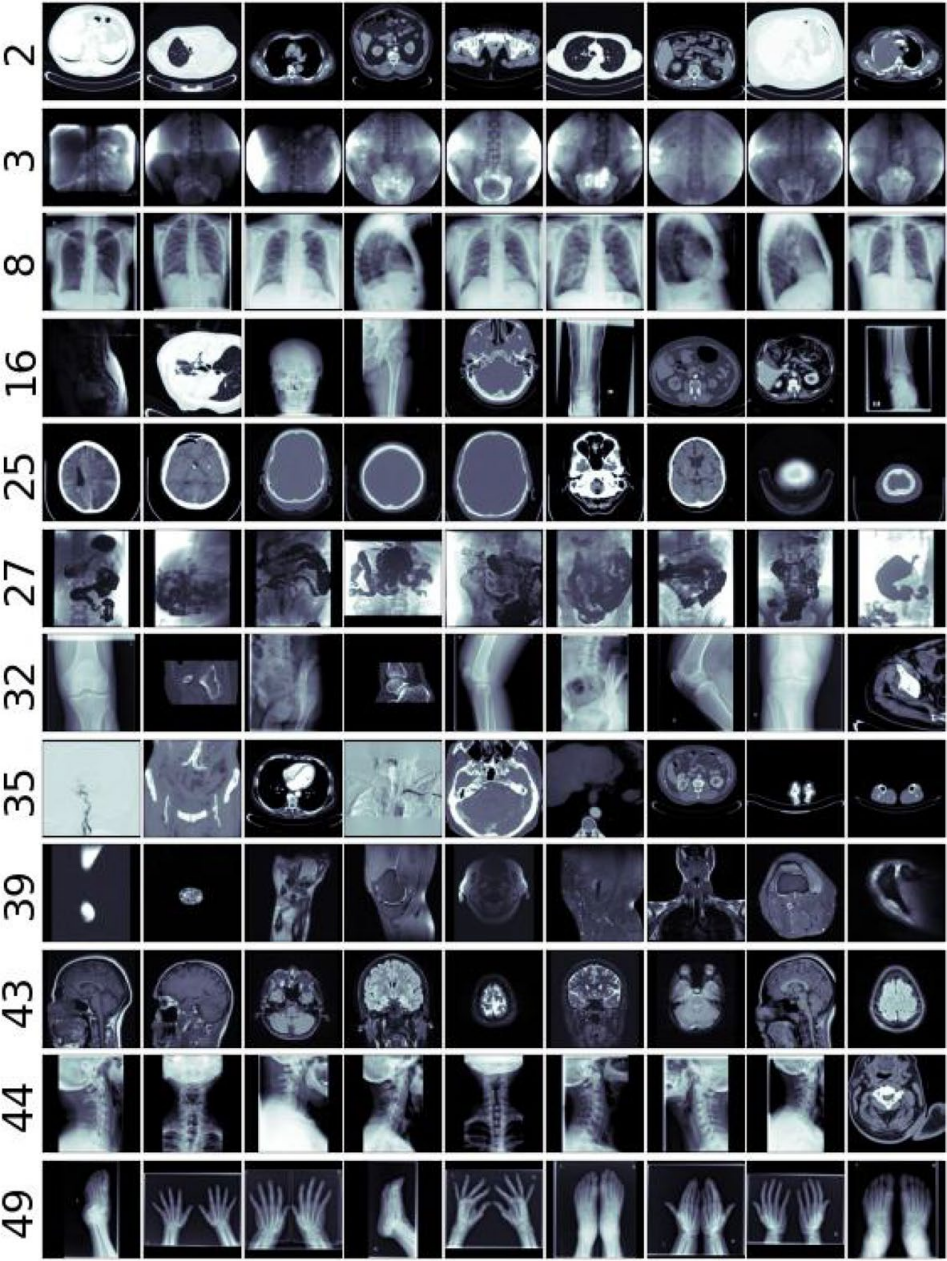
Randomly sampled images from twelve selected clusters. Cluster indices are indicated to the left of each row

**Fig. 9:**
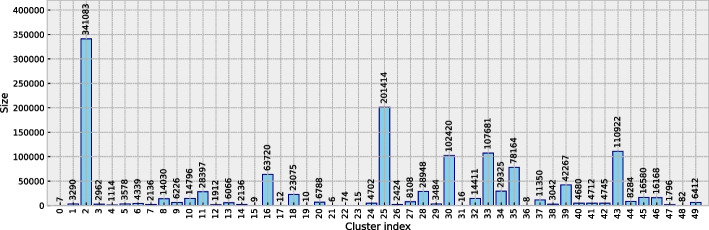
Sizes of obtained groups in the labelled RadiologyNET dataset

**Fig. 10:**
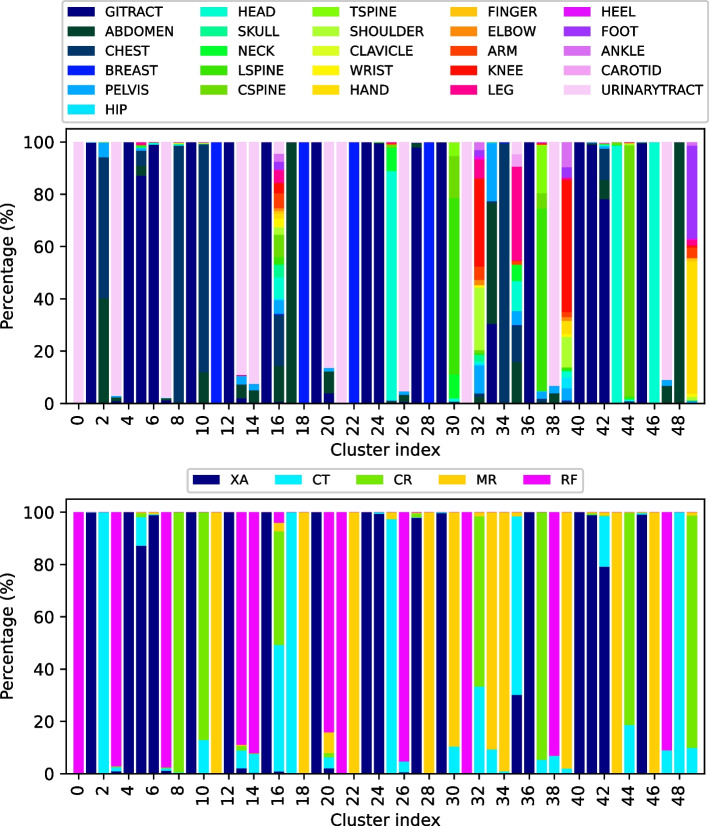
Grouping quality regarding modality and examined body part, for the labelled RadiologyNET dataset. The first image shows how homogeneous the clusters are when observing the body part, while the second one shows the different modalities in each cluster

From the original dataset described in Appendix B, the set of 1,337,926 data points which fit the aforementioned criteria related to image, diagnosis and DICOM tag policies were extracted. The chosen labelling algorithm, illustrated in Fig. 7, was used to cluster this more extensive set of data points into 50 groups. As is shown in Fig. 9, the obtained groups varied in size, with the largest one having 341,083 data points, and the smallest one comprising of only 6 data points. Such small clusters can be considered to contain anomalies which do not fit into other groups. Figure 10 shows the quality of grouping throughout all of the clusters. One can see how the quality of grouping corresponds to the one shown in Fig. 6b), indicating that using new data, which was previously unseen by the labelling algorithm, did not significantly influence the quality of clusters. Groups which were heterogeneous on the smaller set (16, 32, 35 and 39) remained the same after labelling the larger set, and the same applies to homogeneous clusters such as 2, 3, 8, 25, 43 and 44. Random instances of images from these (and other) clusters can be seen in Fig. 8.

Next, regarding the quality of obtained groups presented in Fig. 10, it can be seen that almost all clusters show a high level of homogeneity when considering their imaging modality. On the other hand, body part homogeneity shows how nearby anatomic regions are often grouped together. This is especially prominent in the torso region, where it is difficult to accurately discern the exact border between the abdomen, the gastrointestinal tract and the pelvis (cluster 33), as well as the abdomen and the chest (cluster 2). Also, it is not unusual that a single study contains multiple parts of the torso, for example, an MR image capturing both the abdomen and the pelvis. The same applies to groupings of the spine, where different spinal parts are often assigned to the same group (clusters 30, 37). Images capturing the extremities (hands and feet) were also grouped together in cluster 49, despite being visually dissimilar from one another and showing different anatomical regions. On the other hand, clusters 16, 32, 35 and 39 showed evidence of containing anomalies; they contained images depicting non-connected anatomical regions (leg, abdomen, head, urinary tract...).

### Clustering quality for future models training

Since the final goal of this work was to build a labelled dataset on which NNs can be trained, clusters of lower quality could present an issue. A low-quality cluster can be either a cluster having only a few instances (such as cluster 6 in Fig. 9), or a cluster having high heterogeneity (such as cluster 16 in Fig. 10). These low-quality clusters often cause NNs to struggle to converge during training, consequently leading to their poor performance. There are two viable independent solutions to this problem; they are described next.

The first solution is to remove all low-quality clusters by setting the constraint which requires that clusters must have at least 100 data points – as a clustering post-processing step. Additionally, to remove heterogeneous clusters, the entropy of BPE and Modality labels can be calculated and used to form a cut-off threshold. Any cluster having an entropy score $\ge 0.9$, as was the case in clusters 16, 32, 35 and 39, can be eliminated. By imposing these constraints, the number of clusters is reduced to 36. The remaining 36 clusters contain 1,139,125 unique data instances. With the random split of the dataset to train:validation:test subsets in ratio 75 : 12.5 : 12.5, we are left with 854,334 unique data points in the training subset, and with 142,407 unique data points in test/validation subsets. By oversampling (with random plausible augmentation) on the training subset and matching the size of the biggest group, the training subset rises to 9,209,232 non-unique data points. With this amount of data, we believe that models are capable of learning good representations of distinct classes.

The second possible solution is to increase the number of non-empty clusters. By doing this, the data points will be better distributed among clusters, which ultimately creates a more fine-grained annotated dataset, albeit reducing cluster completeness [50]. As a result, it makes the target problem for NNs more challenging, forcing them to develop more versatile filters and enhance their robustness. This is evident from the ImageNet dataset [7], which includes 1,000 classes. To expand the number of clusters, it is possible to utilise more advanced clustering algorithms such as hierarchical clustering, which is more flexible and intuitive than k-means algorithm [54]. However, advanced clustering algorithms also increase computational complexity, so they were not considered in this research (though they will be considered in the next iteration of RadiologyNET). Another way to increase the number of clusters is to change the data point embedding based on which clustering is done. This requires experimenting with different feature extraction algorithms to achieve richer embeddings from the data sources (for instance, BERT and GPT for diagnoses [35–37]). Further enhancement of embeddings could be achieved by combining three source embeddings more complexly by introducing constraints, e.g. contrasting semantically similar and dissimilar pairs of embeddings [55]. As mentioned before, these potential enhancements are set as future work of our research.

## Conclusion

This paper addresses the challenge of building a sizeable annotated dataset of medical radiology images, using unsupervised machine learning methods to discover useful patterns by combining three data sources: DICOM metadata, images and narrative diagnoses. The purpose of the dataset is to create an ImageNet counterpart for training standard deep learning classification model architectures tuned to medical radiology imaging tasks. Obtained clustering rules exhibit good homogeneity regarding the imaging modality and the anatomic region on a representative data subset.

The final evaluation of the RadiologyNET annotation system will involve assessing TL models on open challenges in medical radiology image processing. This testing will provide a comprehensive and unbiased evaluation of the system’s performance and enable a direct comparison with other state-of-the-art TL techniques; and will also assess the system’s robustness, accuracy, and how well it can generalise problems. One thing that must be pointed out is the possibility of reduced TL performance due to the distribution shift and the currently limited number of distinct classes. While we aim to provide comprehensive fine-grained coverage of various imaging modalities and protocols, it is important to note that not all modalities and protocols are currently included in the annotated dataset, nor is the number of distinct visual categories large enough to make it comparable to ImageNet. Hence, these limitations may potentially impact TL efficiency in practical applications.

The described approach is fully unsupervised, whereas using supervised methods remains a topic of future work. Leveraging modality and examined body part to train supervised feature extractors could possibly benefit the overall quality of the labelled dataset. On another note, textual diagnoses proved to be efficient in grouping medical images, achieving a high *S* score and having the resulting cluster be homogeneous regarding the anatomical region they are depicting. This work could be expanded upon by exploring sophisticated natural language processing (NLP) methods such as GPT or BERT and using sentence encoders to get more accurate diagnoses embeddings. The described extension, along with advanced image and DICOM feature extractors, will unlock the possibility of generating a larger number of clusters, which would be beneficial for model pretraining.

In summary, in addition to documenting the preliminary creation of the RadiologyNET dataset, this study provides insights into automating the labelling process of DICOM data, highlighting the challenges and achievements in grouping them based on anatomical region and imaging modality. The findings contribute to a better understanding of the limitations and potential improvements in automated labelling algorithms for medical imaging datasets. Future research can build upon these findings to refine and enhance the grouping process, ultimately aiding in more accurate and meaningful analysis of medical images.

## Figures and Tables

**Fig. 11:**
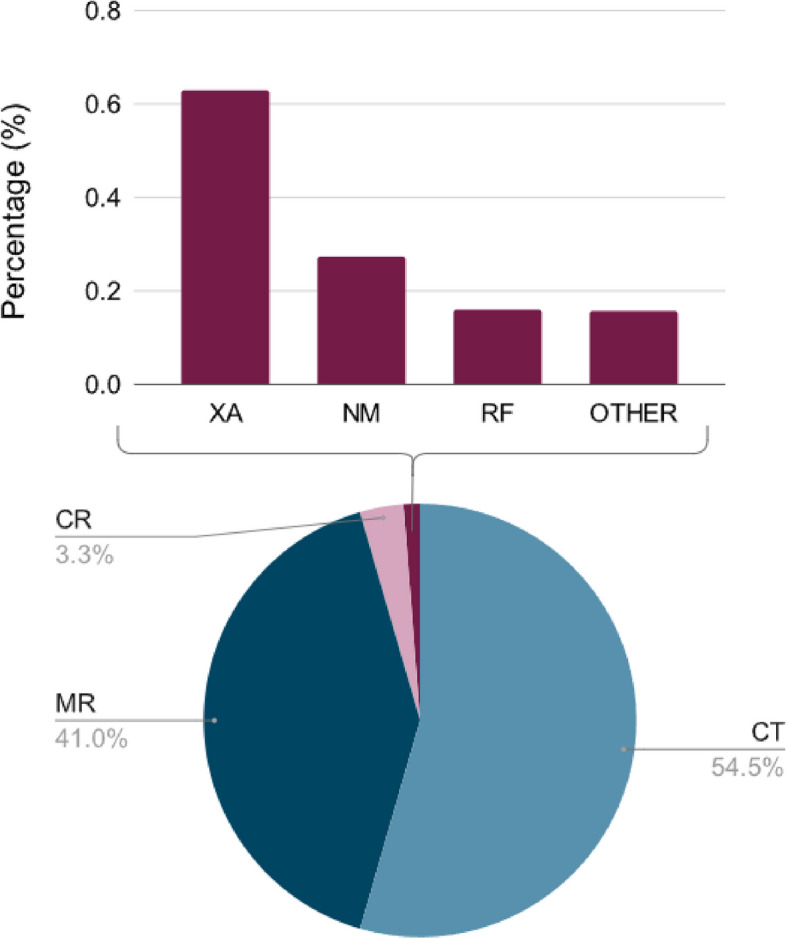
Distribution of modalities across the entire 25 million DICOM files

## Data Availability

No datasets were generated or analysed during the current study.

## Variable description

Variables used in the text are listed and briefly described in Table 8.

**Table 8:** A list and a brief description of each variable used in the text

Variable name	Variable description
$\kappa$	Number of clusters.
*hat* *y*	Predicted cluster label
$hat{y}^{(i)}$	Predicted cluster label of data point *i*
$y^{(i)}_t$	Observed target variable *t* value of data point *i* ($y_{B}$ or $y_{M}$).
$hat{y}^{(i)}_t$	Predicted target variable *t* value of instance *i* ($\hat{y}_{B}$ or $\hat{y}_{M}$).
$x^{(i)}$	Input data instance *i* (all sources).
$x^{(i)}_{I}$	Input data instance *i* image.
$x^{(i)}_{T}$	Input data instance *i* DICOM tags.
$x^{(i)}_{D}$	Input data instance *i* diagnosis.
$f\left( x^{(i)}\right)$	Input data instance *i* embedding (all sources).
$f\left( x^{(i)}_{I}\right)$	Input data instance *i* image embedding.
$f\left( x^{(i)}_{T}\right)$	Input data instance *i* DICOM tags embedding.
$f\left( x^{(i)}_{D}\right)$	Input data instance *i* diagnosis embedding.
$NMI_M$	NMI regarding Modality.
$NMI_B$	NMI regarding BodyPartExamined.
$HS_M$	HS regarding Modality.
$HS_B$	HS regarding BodyPartExamined.
*S*	The harmonic mean of $HS_B$, $HS_M$, $NMI_M$ and $NMI_B$ which will be used to select diagnosis/image models.
$D_I$	The mean distance of image embeddings across all clusters.
$D_D$	The mean distance of diagnoses embeddings across all clusters.
$D_{score}$	The harmonic mean of $D_I$ and $D_D.$

## Extraction of experimental data subset

The material presented here contains the details that complement the text in “Experimentation dataset” section. The dataset consists of approximately 2 million unique exams completed between 2008 and 2017 and performed through standard clinical practice at Clinical Hospital Centre (CHC) Rijeka. It was gathered retrospectively in 2017. The data was anonymised during the extraction process to remove all sensitive information. We obtained approval from the competent Ethics Committee to collect and process the data for this purpose. The obtained approval mandates that the data remains private in its current form.

Each exam could result in a respective diagnosis and at least one (often more than one) DICOM file, which was then stored at CHC Rijeka’s Picture Archiving and Communication System (PACS). The total number of DICOM files obtained from CHC Rijeka PACS reached approximately 25 million [56]. The most prevalent modalities in the dataset were CT, MR, XA, NM (Nuclear Medicine) and RF, as is shown in Fig. 11.

Not all exams resulted in a recorded diagnosis, meaning that diagnoses of some of the performed exams were empty or null. This was the case in roughly $6.96\%$ of data, while $93.03\%$ contained a non-null, non-empty value. Upon further inspection, we discovered that some of the non-empty diagnoses were less than 5 characters long. These were presumed to be anomalies as such short diagnoses could seldom carry useful information. Empty diagnoses and diagnoses which had less than 5 characters were excluded from the used dataset.

From the original dataset we sampled a subset of 135, 775 DICOM files and adjoined textual diagnoses. During the sampling process, special attention was given to include as many complete diagnoses as possible. Namely, an analysis of the available data revealed that there were examinations which resulted in more than 1000 different DICOM files due to multiple projections or views used by radiologists. To avoid a disproportion between the number of distinct diagnoses and DICOM files in the subset, a threshold of 15 files was chosen. This means that all exams which resulted in more than 15 DICOM files were eliminated outright. The sampled subset contained images acquired in 5 different modalities: CT, MR, CR, XA and RF. Although initially included in the subset, images recorded in the NM modality were removed because they were often associated with examinations whose diagnoses were empty, short, or otherwise uninformative.

## DICOM Tags extraction and imputation

The material presented here, where we address several groups of problems related to DICOM tags, contains the details that complement the text in “DICOM tags” section. The first encountered problem was **DICOM tags with missing BPE**, which contained an empty value in $59.4\%$ cases. On the other hand, tags such as *ProtocolName*, *StudyDescription* and *RequestedProcedureDescription* faired better, having empty values in only $10.9\%$, $7.39\%$ and $33.8\%$ of instances, respectively. Wherever *BodyPartExamined* was empty, at least one of the mentioned tags contained a value from which one can infer the examined body part, which is why these three particular tags were chosen. In order to solve the missing values for *BodyPartExamined* tag, there were 53 regular expressions written, which contained rules for imputing *BodyPartExamined* from the *ProtocolName*, *StudyDescription* and *RequestedProcedureDescription* tags. These were also written in a way that account for possible typographical errors (e.g. *torax* and *thorax*), multiple languages used by physicians (e.g. Latin: *calcaneus*; English: *heel bone*; and Croatian: *petna kost*), possible abbreviations (e.g. *c-spine*, *c_spine*, *cspine* and *cervical spine*), and which procedures impact which body part (e.g. *chemoembolization* is tied to the liver, which is a part of the urinary tract). These rules were written under a radiologist’s guidance, as there is no straightforward ruleset for perfect BPE mapping.

The final result of BPE imputation (based on knowledge, decision rules, and regular expressions) was manifested in a jump from $40.6\%$ to $100\%$ non-empty instances. However, we should note that there was still a possibility of erroneously imputing *BodyPartExamined* from other tags. Specifically, DICOM tags *StudyDescription*, *ProtocolName* and *RequestedProcedureDescription* are input manually by a performing physician. As such, other than typographical errors, it is possible that other types of errors could lead to mislabelling of a body part that was not accounted for. However, these cases were presumed to be anomalies and only present in a few DICOM files.

The next group of tags needing additional care are stringified arrays - **DICOM tags with multiple values**. Namely, DICOM tags can contain multiple values, for example, *ImageType* and *WindowCenter*. Such tags were parsed from a single stringified array-like tag into multiple tags, which resulted in *ImageType* dissolving into *ImageType0* and *ImageType1*, etc.

Another group of DICOM tag problems was **selection of appropriate DICOM tags**. There were 654 different DICOM tags that appeared at least once in the whole subset. However, many proved to be uninformative due to either being empty in most instances or having only one distinct value. A fill rate threshold was imposed on each tag, and each tag with less than $35\%$ non-empty values was removed. Furthermore, all DICOM tags with less than 2 distinct values were discarded, along with tags containing unique identifiers, such as *SOPInstanceUID*. After this, continuous and categorical DICOM tags were separated, and categorical variables were further examined. In particular, some of the tags contained natural language, which fell out of the scope of DICOM tag processing. The eliminated tags include the aforementioned *ProtocolName*, *StudyDescription* and *RequestedProcedureDescription*, accompanied by *AdmittingDiagnosesDescription*, *ImageComments*, etc. The remaining categorical variables had no more than 50 unique values. After this, 55 tags remained, of which 28 were continuous and 27 were categorical variables.

The final problem to solve regarding DICOM tags was **missing data analysis**. As was mentioned before, BPE can be directly imputed from other tags via regular expressions, but other values’ imputation is not as straightforward. Before imputing these values, the DICOM tags with missing data were analysed further. To determine if data were missing-completely-at-random (MCAR) or missing-at-random (MAR) [22, 23], univariate statistical tests were performed as described by Enders [21]. Statistical tests differed based on whether the observed variable was discrete or continuous and, in the latter case, if it was normally distributed. If a continuous variable was normally distributed (Shapiro-Wilk, $p\ge 0.05$), then an Independent t-test was performed, while a Mann-Whitney U was applied otherwise. In the case of categorical data, a $\chi ^{2}$ (chi-square) test was used. A variable would not be considered MCAR if its missingness influenced the distribution of at least one other variable, i.e., there was a statistical difference in the distribution where said variable was missing versus where it was not. Although the used approach has its drawbacks [21], it can bring attention to dependencies between variables. Furthermore, consulting with the DICOM standard [15, 57] strengthens the assumption that data is not MCAR. The missing values were imputed using MissForest [23, 24], which had been previously shown to work well with MAR data [25]. MSE was used as the criterion for continuous and Gini impurity for categorical variables. After imputation, the categorical variables were one-hot encoded, and continuous variables were scaled to fit the range [0.00, 1.00]. We performed a small-scale experiment to observe the influence of MissForest imputation versus simple imputation with the mean value [27] and observed better scores related to anatomical region grouping.

## Image export from DICOM

The material presented here contains the details that complement the text in “Images” section. In order to export images from DICOM files, several parameters are required to be present among the DICOM tags. The initial parameters/tags that require verification are the image (*PixelData* DICOM tag) and modality (*Modality* DICOM tag). Namely, some modalities demand scaling of the raw image pixels’ values to values that are meaningful to the targeted application (emphasizing the region of interest for the radiologist). Scaling of each raw image values $x_{Ir}$ is performed using the expression $x_I'= R_s \cdot x_{Ir} + R_i$, where $x_{Ir}$ and $x_I'$ are raw pixel values (the input image) and the rescaled pixel values, respectively; $R_s$ is the rescale slope, and $R_i$ is the rescale intercept. *RescaleSlope* and *RescaleIntercept* are DICOM tags located in the DICOM metadata [15].

If DICOM does not contain values for $R_s$ and $R_i$, the default values $R_s = 1$ and $R_i = 0$ are used. In situations where the transformation was not linear, we did not use the look-up table (LUT) for transformation [58]. We would drop the DICOM in these cases because implementing LUT is case-dependent. The number of cases with LUT in our dataset was negligible, and we could afford to drop the data.

Next, pixel values must be mapped to 8-bit resolution, i.e., interval [0, 255]. The selection of which values will correspond to 0 and which to 255 relies on the specific modality and the organ/tissue being examined. To correctly map pixel values to the 8-bit range, we have followed established practices in radiology which can be formulated as follows [59]:

6 $$\begin{aligned} x_I = \left\{ \begin{array}{ll} 0, &{} \text {if } x_I' \le W_l \\ 255, &{} \text {if } x_I' \ge W_u\\ \frac{1}{W_w} (x_I' - W_c + \frac{1}{2}W_w) \cdot 255 , &{} \text {otherwise}\\ \end{array}\right. \end{aligned}$$

where $W_l$ and $W_u$ are lower and upper window boundaries, respectively; $\hat{x}_I'$ is the image received after applying rescale slope and intercept; and $x_I$ is the exported 8-bit image. $W_l$ and $W_u$ are calculated as:

7 $$\begin{aligned} W_l = W_c - \frac{1}{2}W_w, \hspace{1cm} W_u = W_c + \frac{1}{2}W_w. \end{aligned}$$

The parameters *WindowCenter* ($W_c$) and *WindowWidth* ($W_w$) are read out from the DICOM metadata. If multiple values were supplied for *WindowCenter* and *WindowWidth*, as is sometimes the case in modalities such as XA, only the first valid value was used. The windowing parameters were considered valid if the resulting image was not entirely single-coloured (e.g. completely black or completely white). Also, since some imaging techniques like MR can have multiple slices, we decided only to use the first slice, which contained meaningful data. The meaningful data was selected on two introduced policies.

The *value policy* was introduced to filter out images containing little-to-no information. *Value policy* calculates the ratio $r_V$ between the number of different pixel values in the image and the total possible number of different pixel values in an image. The calculated ratio $r_V$ was required to be higher than $t_V = 0.1$. Threshold $t_V$ was experimentally determined.

On the other hand, the *shape policy* demanded that the ratio $r_S$ between exported image width and height was higher than threshold $t_S = 0.1$. Threshold $t_S$ was experimentally defined. By applying this *shape policy*, all erroneous shapes (such as vectors) were filtered out.

To summarise, the raw pixel data is first transferred to the desired pixels’ intensity range with the *RescaleSlope* and *RescaleIntercept* parameters. Then, the intensity range of the pixels needs to be converted to the 8-bit range, which is accomplished by utilising the *WindowCenter* and *WindowWidth* parameters. Finally, it is necessary to check if the content is informative for certain modalities. This is achieved by applying *value* and *shape* policies.
